# Rational Design of Peptide-Based Inhibitors Disrupting Protein-Protein Interactions

**DOI:** 10.3389/fchem.2021.682675

**Published:** 2021-05-04

**Authors:** Xuefei Wang, Duan Ni, Yaqin Liu, Shaoyong Lu

**Affiliations:** ^1^State Key Laboratory of Oncogenes and Related Genes, Shanghai Jiao-Tong University School of Medicine, Shanghai, China; ^2^The Charles Perkins Centre, The University of Sydney, Sydney, NSW, Australia; ^3^Medicinal Bioinformatics Center, Shanghai Jiao-Tong University School of Medicine, Shanghai, China

**Keywords:** protein-protein interaction, undruggable, peptide, peptidomimetics, drug discovery

## Abstract

Protein-protein interactions (PPIs) are well-established as a class of promising drug targets for their implications in a wide range of biological processes. However, drug development toward PPIs is inevitably hampered by their flat and wide interfaces, which generally lack suitable pockets for ligand binding, rendering most PPI systems “undruggable.” Here, we summarized drug design strategies for developing peptide-based PPI inhibitors. Importantly, several quintessential examples toward well-established PPI targets such as Bcl-2 family members, p53-MDM2, as well as APC-Asef are presented to illustrate the detailed schemes for peptide-based PPI inhibitor development and optimizations. This review supplies a comprehensive overview of recent progresses in drug discovery targeting PPIs through peptides or peptidomimetics, and will shed light on future therapeutic agent development toward the historically “intractable” PPI systems.

## Introduction

Protein-protein interactions (PPIs) play a fundamental role in all life events and cellular activities, regulating cells' lives and death, as well as mediating various biochemical reactions like signal transduction and metabolisms (Li et al., 2020; Luck et al., 2020; Thanasomboon et al., 2020). Thus, PPIs are regarded as the “holy grail” of modern life science and medicine, and they have emerged as a class of promising therapeutic targets toward a plethora of medical conditions (Cunningham et al., 2017; Davenport et al., 2020). PPIs can make up a large-scale and complicated network termed as “interactome” (Koh et al., 2012). Current studies estimated that human interactome consists of about 650,000 PPIs, which represents a fruitful repertoire of drug targets for therapeutics discovery (Vidal et al., 2011). Modulating PPIs is of critical significance in both basic research and clinical translations. It not only facilitates our better understanding to a wide range of biological events, but also constitutes the theoretical basis for current therapeutic agents development. In the era of modern pharmacology, rational design of PPI inhibitor is considered to be a prospective direction for drug discovery, and possess enormous potentials.

Historically, PPIs have attracted extensive attentions in their related researches (Tsai et al., 2009; Milroy et al., 2014; Devkota and Wuchty, 2020). Enormous efforts invested on this topic have retrieved a plethora of high quality PPI system crystal structures, based on which some success has been accomplished in the PPI inhibitor drug design (Schmidt et al., 2014; Sledz and Caflisch, 2018). Nevertheless, due to the by nature biophysical and biochemical limitations, drug discovery targeting PPIs still remains a tough task in both academia and industry. One of the utmost difficulties for PPI inhibitor development is the large, shallow and featureless PPI interfaces. Such poor surface architecture poses considerable difficulty for ligand binding, as well as confers great challenges toward the design and optimizations of drug molecules (Ni et al., 2019). Previous crystallography and modeling studies have unveiled that, in stark contrast to the small molecule binding site, which is relatively deep and covers only 300–500 Å^2^ area, protein binding interface is generally wide and flat, and its surface area ranges approximately between 1,000–2,000 Å^2^ (Lo Conte et al., 1999; Ran and Gestwicki, 2018). Consequently, given the difficult topologies of PPI systems, they are commonly deemed as “undruggable,” with limited specific inhibitors directing to them identified. However, the recent proposal of “hot spot” notion has greatly promoted the development of PPI inhibitors (Bogan and Thorn, 1998). It provides a tempting alternative for designing orthosteric ligands to directly target the interaction interface, especially for developing motif-based peptide inhibitors, which mimic the important secondary structure, i.e., the “hot spots” along the PPI interface and disrupt its formation.

The last decade has witnessed remarkable advances in handling challenging PPI targets with small molecules (Ji et al., 2017; Chen et al., 2018). Notably, venetoclax (Souers et al., 2013), which is considered as the first FDA-approved BH3-mimetic drug to interfere with PPI, is a ground-breaking milestone of fragment-based drug discovery (Deeks, 2016). With regard to the development of peptides into drugs, unlike small molecules, they mildly occupy a relatively small portion (~2%) of the global drug market (Di, 2015). In general, peptide inhibitors support to address some disease targets, which are difficult to be treated with small molecules. Approximately more than 30 peptide drugs were approved during the last two decades (Lee et al., 2019).

Although small molecules dominate the field of drug market so far, peptide inhibitors still represent a class of promising candidates because of their similarity to endogenous ligand, high affinity, and low toxicity. Therapeutic peptides have been validated that they can effectively and selectively inhibit PPIs both in cancer and virus (Lau and Dunn, 2018). On the other hand, drugging PPI targets with peptides or peptide-based inhibitors is also facing various challenges. They are frequently hampered from progressing into clinic owing to their low membrane permeability, poor oral bioavailability and short half-lives after administration (Di, 2015). Additionally, how to achieve higher affinity and better synthesis diversity are also long-held conundrums for peptide-related drug molecule rational design. Rapid advances in medicinal chemistry and chemical biology have proposed a panel of promising resolutions to tackles these issues, including cyclic peptides, hydrogen bond surrogate, and stapled peptides, which can optimize the ADME (Absorption, Distribution, Metabolism, and Excretion) properties of the peptide-based compounds (Sohrabi et al., 2020). Furthermore, strategies such as introduction of rigid backbone linkers and addition of unnatural side chains or modified moiety can also help to enhance ligand affinity and chemical diversity, contributing improved druggability (Klein et al., 2014; Stevenazzi et al., 2014). Notably, these fast-progressing techniques have already obtained reasonable success in an increasing number of critical targets, such as Bcl-2 family members, p53-MDM2, as well as APC-Asef, exhibiting great prospects for future PPI drug discovery and optimizations.

Here, our review first outlines the key characteristics of PPIs and summarizes advanced peptide-based drug candidates. We aim to provide more inspiring insights that will hopefully enhance future PPI drug development. We initially introduce the basic features of proteins binding interface and subsequently give a summary of current methods for designing PPI inhibitors. Importantly, we focus on several successful examples of popular targets to illustrate the feasibility of these modulator design strategies in regulating PPIs. Such cases are holding promise to improve our understanding toward the sophisticated PPI system and aid to accelerate future development of peptide-based PPI inhibitors.

## Characteristics of PPI Systems

There are a large number of PPI complexes in every organism, some of which turn out to be permanently bound, while others are transient and bind or dissociate under different conditions, subject to changes in temperature and pH, etc. In addition to their overall dynamic nature, PPI interfaces also exhibit different biochemical and biophysical properties relative to the small molecule compound binding pockets. Such unique characteristics of PPI complexes and their interfaces confer differential challenges for their modulation, and distinct therapeutics development strategies have thus been proposed accordingly. In this section, we briefly discuss the structural and biological features of PPI interfaces, which underlie the mechanistic basis for drug discovery targeting PPI systems.

### Structural Properties of PPI Interface

Analysis of the 3D structures of PPI complexes indicates that their interfaces are relatively large and shallow, falling within the range of 1,000–2,000 Å^2^, compared to the ones for typical small molecule binding sites for around 300–500 Å^2^. Among them, a “standard size” PPI interface is estimated to be about 1,600 ${Å}_{,}^{2}$ and composed of around 52 amino acid residues (Lo Conte et al., 1999). Historically, PPIs were deemed as “intractable” with their large and flat binding surfaces being smooth and lacking well-defined binding pockets. Furthermore, the high affinity and tight interactions between PPI partners also posed great challenge for ligands to compete for binding orthosterically, hampering the design of PPI inhibitors. Nevertheless, the ever-developing structural analyses have contributed to the emergence of the concept—“hot spots,” which has considerably challenged the primitive notion that PPIs are “undruggable” (Bogan and Thorn, 1998). Along the PPI interface, if a residue mutated to alanine with the PPI binding energy differing for more than 2 kcal/mol, it is defined as a hot spot. The area of hot spots are considered to be only about 600 Å^2^ (Lo Conte et al., 1999). It is validated that hot spots residues mainly distribute in the center of the PPI interface, and they frequently consist of Trp, Tyr, and Arg (Halperin et al., 2004). The existence of hot spot residues has thus shed light on PPI inhibitors development as it supplies a more specific and well-defined drug target instead of the broad and wide protein interaction surfaces. Through interfering with the hot spot residues within a relatively local region, inhibitor molecules could readily avoid competing with the high affinity protein binding effector while easily disrupting the overall PPI complexes and exerting therapeutic effects. Hence, in summary, understanding PPI structures, especially the corresponding hot spot residues are of vital significance to PPI drug design. So far, targeting PPI hotspots has achieved reasonable success in PPI drug discovery (Zerbe et al., 2012; Akram et al., 2014). In general, medicinal chemists would first focus on the proteins binding interfaces obtained through crystallography studies. Through exhaustive alanine mutagenesis screening or computational analyses (Lao et al., 2014), hotspot residues could be identified and next aid to instruct structure-based inhibitor design. To mimic hotspots' structural effects implicated in PPI processes, various compound candidates are subsequently screened and rationally modified. Through conformational investigations and structure activity relationship analyses and other pharmacological refinements, the potency of the hotspot-based inhibitors can be further optimized, marking the success of the hit-to-lead optimization workflow. Following such scheme, researchers have managed to design a myriad of PPI inhibitors toward a plethora of historically intractable targets including the immune responses-related CD2-CD58 (Liu et al., 2005) and PD-1-PD-L1 (Lim et al., 2019; Yang and Hu, 2019), inflammation-related TLR4-MD2 (Liu et al., 2011) and apoptosis-related Mcl-1-Bim (Denis et al., 2020). Their success depicts the importance of PPI hotspot elucidation and the downstream structure-guided PPI inhibitor discovery.

### Biochemical Properties of PPI Interface

In addition to the structural features, biochemical properties of PPI systems such as their complementarity and hydrophobicity are also critical factors to consider in designing PPI inhibitors (Chene, 2006; Isvoran et al., 2013). In a PPI complex, complementarity is defined as a particular condition that involves the matching of the surface residues from the corresponding interaction partners. Interfaces with less complementarity possess weaker binding force and therefore can be more readily disrupted by inhibitor molecules. From previous studies, it was demonstrated that the complementarity of both homodimer and permanent complex is considerably stronger than that of heterocomplex and non-permanent complex (Xiao and Konermann, 2015). On the other hand, the hydrophobic degree is mainly characterized by the amount of water molecules on the interface. Retrospective studies manifested that the protein-protein interface is predominantly occupied by 56% non-polar groups, 29% polar groups, and 15% charged groups, respectively (Chene, 2006). It also shows the trend that aromatic residues and hydrophobic residues have higher propensities for interfaces, while hydrophilic residues are more preferred in the buried portions. Thus, it is conceivable that hydrophobicity, polarity and other chemical properties of inhibitors are critical factors to consider for their binding and targeting.

## Drug Design Methods of Peptide-Based PPI Inhibitors

PPI inhibitors can mainly be classified into three directions, i.e., small synthetic molecule inhibitors, peptide inhibitors and peptidomimetics inhibitors. By nature, small molecules frequently suffer from undesirable selectivity, resulting in off-target side effects (Lin et al., 2019), whereas peptides commonly exhibit affordable synthesis availability and more favorable specificity for their targets. Most noteworthy is the additional chemical modifications for synthetic peptides, which are typically designed to mimic key structural domains in the natural PPI partners. Peptide-based inhibitors, with increased bioavailability and improved permeability, have been gaining prominence in the field of PPI drug design.

Here, we discuss the advanced strategies for the development of peptide-based inhibitors in terms of peptides and peptidomimetics.

### Design Method for Peptide Inhibitors

The conventional method of designing peptide inhibitors is to introduce tailored oligopeptides that are modified based on several important residues along the original PPI interfaces (e.g., hot spots) to inhibit proteins binding (Schatti et al., 2017). It is worth noting that the design of peptide inhibitors efforts have made impressive progress over the past years (Scott et al., 2016). Ya-Qiu Long and colleagues designed a series of short peptides (7–17 residues) comprising key amino acid residues which are critical for integrase (IN) catalytic activities or viral replication (Li et al., 2006). They presented a novel “sequence walk” strategy covering the whole 288 residues of IN in an attempt to unravel the “hot spots” of PPI on IN. Two novel peptides NL-6 and NL-9 were then identified with IC_50_ values of 2.7 and 56 μM for strand transfer activity. In addition, Mingjie Zhang group has discovered a potent and specific GABARAP-selective peptide targeting Atg8-AnkG to inhibit autophagy (Li et al., 2018). The highly effective inhibitory peptide derived from 270/480 kDa ankyrin-G binds to GABARAP with *Kd* ≈ 2.6 nM. Although some researchers have successfully developed several peptide inhibitors, the pharmacokinetic properties of peptides still substantially suffer from major drawbacks such as poor cellular permeability and high metabolic instability. These issues considerably limit their further optimizations and clinical applications.

### Design Method for Peptidomimetic Inhibitors

Peptidomimetics are defined as peptide-like molecules that encompass amino acid analogs and other chemical moieties with specific pharmacophores (Vagner et al., 2008; Lenci and Trabocchi, 2020). They are designed to mimic the 3D structures of the original PPI binding partners or segments and further compete with them for PPI disruption. Peptidomimetics usually mimic specific PPI interfacial structures such as α- helixes or β-sheets (Wendt et al., 2021), and the addition of functional modifications toward the lead peptide templates can enormously enhance their potency and efficacy. Relative to natural peptides, introduction of medicinal chemical functional groups or artificial pharmacological structures can substantially enhance their inhibitory activity as well as bypass the intrinsic limitations such as poor proteolysis stability or compromised bioavailability (Qvit et al., 2017). Hence, they are considered as one of the state-of-the-art trends in the field of PPI inhibitor drug development. The design of peptidomimetic inhibitors is frequently classified as the category of structure-based drug design (SBDD) (Pelay-Gimeno et al., 2015). Typically, it starts with selecting key residues within the PPI systems as templates, such as identification of the hotspot residues. Next, through rational modifications, the initial template structures are optimized to ensure that the segments devised are able to compete and fill the space occupied by the originally recognized PPI peptide chains (Shin et al., 2017). Finally, the modified peptide molecules are assessed for pharmacological activity and may undergo further optimizations. Peptidomimetics design is currently regarded as an eminently practical and promising approach for discovering peptide-based PPI inhibitors. So far, they have achieved fruitful success in taming series of critical but intractable PPI targets such as the MLL1-WDR5 (Karatas et al., 2013), APC-Asef (Jiang et al., 2017; He et al., 2021), DCN1-UBC12 (Zhou et al., 2018), and APP-Mint2 (Bartling et al., 2021). Some of them have even marched into clinical applications. For example, Nelfinavir, which was launched into the market in 1997 for the treatment of human immunodeficiency virus (HIV) infectious diseases, was contrived by the aforementioned strategy, exemplifying the power and potential of peptidomimetics-based PPI drug discovery (Wlodawer, 2002).

### Chemical Modifying Approaches for Peptide-Based Inhibitor Optimization

**Figure 1:**
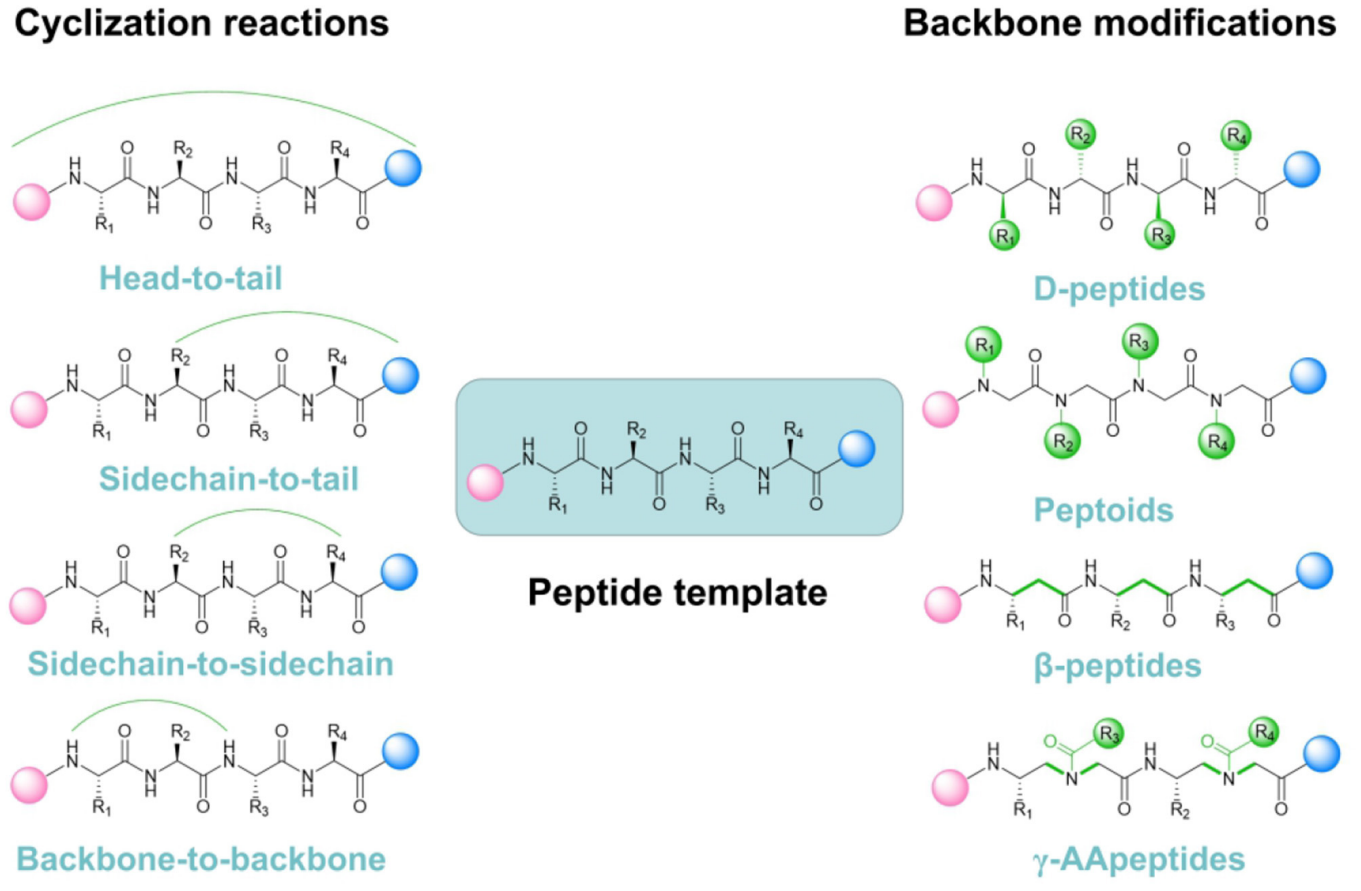
Current representative mainstream strategies for peptidomimetic PPI inhibitors design and optimization.

Improving the stability of the active conformation and reducing its high sensitivity toward proteolysis are among the key challenges when performing peptide-based inhibitor development and modifications. Cyclization and backbone alteration are emerging as two supreme strategies to counteract these problems (Wojcik and Berlicki, 2016) (Figure 1).

The fundamental essence of cyclization is to introduce a rigid structure to augment steric constraints through stabilizing the turns, spirals, and extended conformations within the peptide scaffolds. Various strategies such as hydrogen-bonded substitutes, stapling and hairpins are specifically exploited for such purposes. Understanding and improving peptide-based molecules function through conformational restriction has a long history. Stapling, which means intramolecular side-chain-to-side-chain crosslinking, is proven to be a useful tool to produce peptides with optimized properties (Quartararo et al., 2012). While the β-hairpin motif containing a D-Pro-L-Pro dipeptide template, is also commonly considered as a basic scaffold for PPI inhibitor development (Robinson, 2008). Interestingly, free rotation for molecules with biological activity are available to be restricted by attaching steric effects, introducing disulfide bonds and utilizing chelation of metal ions (Chen et al., 2001). It becomes clear that peptides with rigid conformation retain reduced flexibility, but enhanced selectivity and less toxicity. Bradley L. Pentelute group presented a novel and subtle method for the perfluoroaryl-cysteine SNAr chemistry toward synthesizing macrocyclic peptides via *N*-arylation (Lautrette et al., 2016). Through such approach, they retrieved a potent peptide inhibitor targeting the p53-MDM2 interaction with enhanced proteolytic stability and cell permeability (Touti et al., 2019). With continuous development, researchers also managed to utilize similar approach to develop other macrocyclic low-nanomolar p53-MDM2 interaction inhibitors based on the *i, i* + 4 macrocyclization scheme, which endowed improved membrane penetrating ability and cytotoxicity toward cancer cells, thereby exhibiting more favorable anti-cancer therapeutic efficacy.

Another prevalent trend in peptide-based PPI inhibitor discovery is rational modification based on the structure of peptide backbone. Along such stream, the original peptide chain and the effective functional groups of great importance to the binding site are generally retained, while some relatively unimportant side chain moieties will be subject to rational modifications. This strategy for peptide refinement mostly focuses on alterations in the main chain including introducing D-peptides, β-peptides and peptoids (Lee et al., 2019). Primarily, changing stereochemistry by inserting D-amino acids has emerged as a promising methodology for designing peptides inhibitors. They are more resistant against proteolytic degradation than their L-enantiomeric counterparts (de la Fuente-Nunez et al., 2015). Because L-peptides are usually more easily selected by the chiral proteases and quickly cleaved into corresponding amino acid substituents in blood plasma. Such approach can readily prevent the inhibitor molecules from rapid proteolytic degradation. Seetharama D. Jois and co-workers introduced the changes in the chirality of amino acids in the leading compound to improve metabolic stability (Pallerla et al., 2018). As a result, they successfully designed and synthesized a peptidomimetic containing a cyclic and D-amino acid with a nanomolar level IC_50_ targeting the PPI between HER2 and HER3. Furthermore, adding unnatural amino acids in the main chain to extend the original peptide backbone or replacing peptide bonds with bioelectronic isosteres are also important alternatives to improve the bio-stability of the peptide PPI inhibitors (Choudhary and Raines, 2011). For instance, Aleksandra Misicka and co-workers reported a unique approach that introducing 1,4-disubstituted 1,2,3-triazole rings as peptide bond isosteres to inhibit interaction between NRP-1 and VEGF (Fedorczyk et al., 2019). They managed to modify the “linker” part and the “arm” part of the peptidomimetics to obtain the structural variation. Eventually, the optimized compound Lys (Har)-GlyΨ[Trl]GlyΨ[Trl]Arg showed outstanding resistance toward proteolysis in human plasma as well as displaying favorable IC_50_ value of 8.39 μM.

In addition to the above classical modification methods for sequence-specific peptidomimetics, γ-AApeptides, as an emerging unnatural peptide backbone, have also gradually entered the field of drug leads design directing to PPIs in recent years. It provides more possibilities for the development of peptide-based inhibitors bearing new scaffolds in the exploration of therapeutic agents toward PPIs.

**Figure 2:**
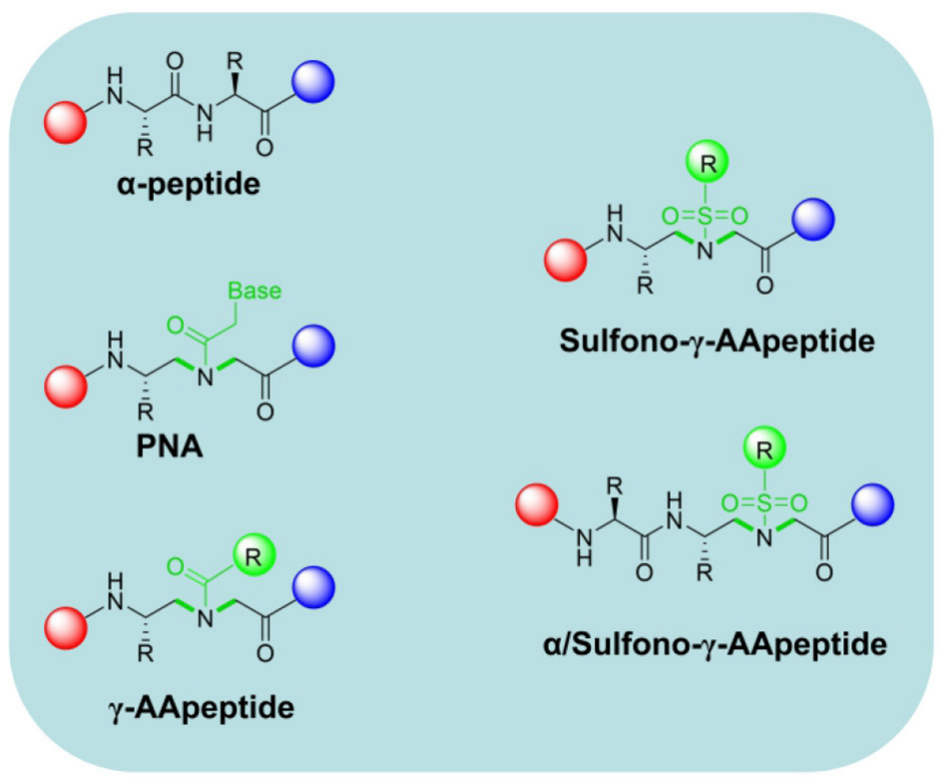
The chemical structure of α-peptide, chiral PNA, γ-AApeptide, Sulfono-γ-AApeptide, and 1:1 α/Sulfono- γ-AApeptide.

γ-AApeptides are constructed based on the chiral peptide nucleic acids (PNAs) backbone (Winssinger et al., 2004; Niu et al., 2011). They are termed as γ-AApeptides since they are oligomers of γ-substituted-*N*-acylated-*N*-aminoethyl amino acids (Figure 2). This series of peptidomimetics are easily carried out on the solid phase. γ-AApeptides can enhance the backbone chemical diversity because half of their side chains could be substituted through the reaction of enormous agents with the secondary amines in the main chain. Both sulfono-γ-AApeptides and 1:1 α/sulfono-γ-AApeptides are investigated for their folding propensity by X-ray, CD, and 2D NMR (Shi et al., 2016; Teng et al., 2016; Nimmagadda et al., 2019). It is supported by 2D NMR analysis that these sequences adopt a helical conformation similar to the α-helix in the solution, and form a myriad of intramolecular hydrogen bonds. Particularly, sulfonyl groups contribute to form more hydrogen bonding, which facilitate the α-helical formation and further stabilize the secondary structure. Cai Group reported a series of unnatural helical sulfono-γ-AApeptides to mimic the conventional α-helix structure, which can effectively and specifically inhibit β-catenin/BCL9 PPI (Sang et al., 2019). They selected the key residues of BCL9 helical domain as the starting point for the development of novel molecular framework, including Arg359, Leu363, Leu366, Ile369, and Leu373. Compared with native BCL9, the foldameric peptidomimetics showed excellent resistance to proteolytic degradation. Furthermore, cellular activity studies manifested that the sulfono-γ-AApeptides are available to penetrate Wnt/β-catenin–dependent cancer cells. These efforts represent a successful application for a new class of helical sulfono-γ-AApeptides in PPI therapeutics.

Herein, we focus on the drug design approach of peptidomimetics and our substantial efforts were to analyze the successful examples reported in the latest literature, hoping that they could contribute a comprehensive overview of peptide-based drug studies.

## Examples of Peptide-Based PPI Inhibitors

### Inhibitors of Bcl-2 Family PPIs

The Bcl-2 protein family is involved in the regulation of cell apoptosis essentially by their members' direct binding interactions that control mitochondrial outer membrane permeabilization (MOMP) (Adams and Cory, 2018; Kale et al., 2018). The Bcl-2 family can be divided into two categories, some proteins promoting cell death like Bax and Bak, while others inhibiting programmed cell death, such as Bcl-xL, Bcl-2, Bcl-w, Mcl-1, and Bfl-1. Bcl-2 family proteins share four homology regions designated the Bcl-2 homology (BH) domains, i.e., BH1, BH2, BH3, and BH4 (Chittenden, 2002; Ashkenazi et al., 2017). Importantly, BH1 and BH2 domains are important regions of death-inhibiting proteins with anti-apoptotic activity and heterodimerization. In stark contrast, the BH3 domain, which exists in all proteins in the Bcl-2 family, is crucial to promote apoptosis (Knight et al., 2019).

The BH3 domain constitutes the structural basis for two mainstream peptide inhibitor design methods toward the Bcl-2 protein family. One is developing small molecule inhibitors mimicking the key BH3 hotspot residues thereby disrupting the related PPIs (Kotschy et al., 2016), while the other is structural modifications based on the BH3 peptide scaffold to retrieve peptide-based PPI inhibitors with improved pharmacological properties (Ryan and Davids, 2019).

**Figure 3:**
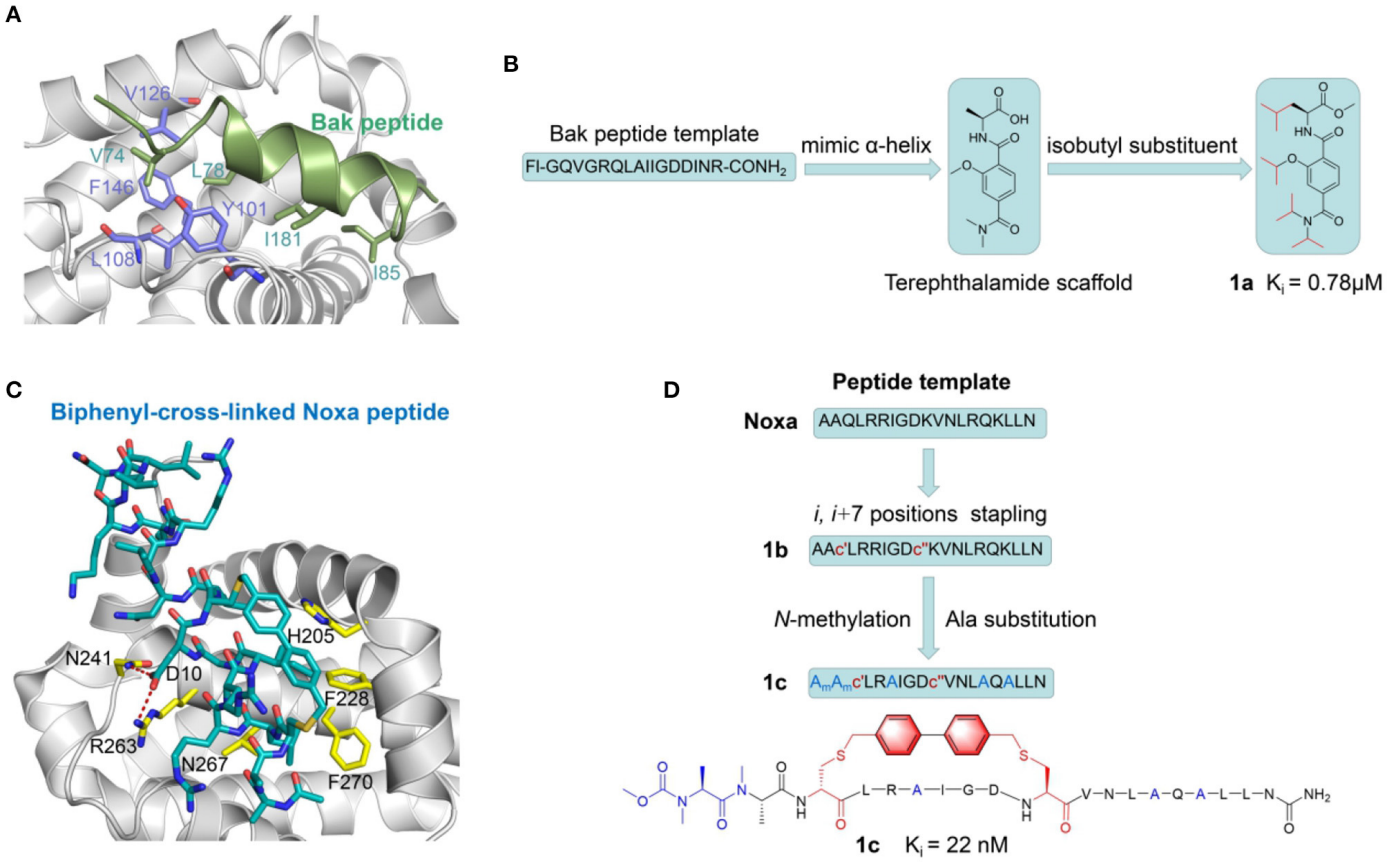
Peptide-based PPI inhibitors targeting Bcl-2 Family PPIs. **(A)** Structural overview of the Bcl-xl/Bak peptide PPI complex (PDB ID: 1bxl). Bcl-xl and Bak are shown in gray and green, respectively. Hydrophobic residues along the PPI interface are highlighted in purple (Bcl-xl) and green (Bak peptide) stick correspondingly. **(B)** Development scheme of Terephthalamide derivatives as BH3 mimetics peptide PPI inhibitors. **(C)** Binding interactions between Biphenyl-cross-linked Noxa peptide (blue carbon atoms) and Mcl-1 (gray carbon atoms). The key residues of side chains in Mcl-1 are labeled in yellow. The red dashed lines represent the hydrogen bonds between Biphenyl-cross-linked Noxa peptide and Mcl-1. **(D)** Development scheme of Noxa stapled peptides as Mcl-1 inhibitors.

One of the classic examples of BH3-based PPI inhibitor is its application in Bcl-xL-Bak PPI system. Based on X-ray crystallography and NMR spectroscopy study, it was shown that Bcl-xL encompasses two central hydrophobic α helices surrounded by five amphipathic helices (Sattler et al., 1997). Further solution NMR experiments revealed that during the Bcl-xL–Bak interaction, Bak binds into a hydrophobic groove on the surface of Bcl-xL using an amphipathic α-helix which consists of hydrophobic side chains including the Val74, Leu78, Ile81, and Ile85 residues (Figure 3A). The structure of Bcl-xL in complexed with Bak peptide containing 16 residues derived from the BH3 region elucidated that this 16-amino acid peptide could be a promising starting point for inhibitor design. Synthetic scaffolds with structural characteristics of the BH3 helix region is proven to be a prospective strategy for developing Bcl-xL–Bak PPI inhibitors. α-Helix mimics derived from a terephthalamide scaffold was selected to mimic the discontinuous binding epitopes of the Bak peptide (Yin and Hamilton, 2004) (Figure 3B). The designed compound **1a** (*Ki* = 0.78 μM) inhibiting the BH3 domain disrupts the Bcl-xL–Bak complex with a comparable affinity to the peptide. The selected terephthalamide scaffold directly mimics the α-helical region of BH3 domain in pro-apoptotic Bak and provides insight into further investigation for rigid small molecules against Bcl-xL–Bak.

Despite their wide application, BH3 mimetic PPI inhibitors inevitably suffer from poor pharmacological properties (Touzeau et al., 2018). It is thus of intensive interest to academia to introduce constrained structure to rigidify peptides in their active conformations, pursuing for enhanced inhibitory activity. Walensky et al. (2004) exploited the hydrocarbon stapling strategy to obtain stapled peptides, which is named “stabilized alpha-helix of BCL-2 domains (SAHBs).” α,α-disubstituted unnatural amino acids with olefin-bearing tethers were used to generate hydrocarbon-stapled peptides by ruthenium catalyzed olefin metathesis. SAHB_A_ simulating the BH3 domain of BID exhibited the high affinity of *Kd* = 38.8 nM compared to that of original BH3 peptide with more than six-fold improvement. In addition, lower molecular weight to promote bio-availability is another direction to improve the drug potential of peptide-based inhibitors. David P. Fairlie group have made an attempt to downsize the BAD BH3 domain based on long stapled peptides (Shepherd et al., 2016). They successfully cut down the BAD BH3 domain to 8–14 residue peptides but still with appreciable affinity for Bcl-xL for PPI disruption.

Subsequent studies presented a panel of Bcl-2 family PPI inhibitors generated by side-chain cross-linking (Beekman et al., 2017). The first crystal structure of inhibitors among this class was reported for a biphenyl-cross-linked Noxa peptide in complex with its target Mcl-1 was determined to provide the structural insights to constructing selective Mcl-1 Inhibitors (Muppidi et al., 2012) (Figure 3C). Inhibitors derived from the sequence of the Mcl-1 BH3 motif were subsequently remodeled the surface by introducing side-chain replacement and *N*-methylation. They previously proposed a notion called dicysteine-mediated cross-linking chemistry, which applies distance-matching bisaryl cross-linkers to generate reinforced peptides and improve their cellular uptake. Following the strategy, researchers replaced *i* and *i*+7 residues (Gln-77, Lys-84) with two cysteines on the basis of the lead structure of NoxaB- (75–93)-C75A peptide and introduced biphenyl-cross-linker into the initial 19-mer peptide. Based on the aforementioned crystal structure, they determined key residues and finally obtained a series of optimized cross-linked peptides. The best peptidomimetic **1C** showed a more than 30-fold increase in inhibitory potency and more preferable cell permeability as well as proteolytic stability (Figure 3D). This example highlights the significant application prospects of cross-linking and complementary peptide modification for future development of potential PPI therapeutics.

Library screening is another promising way to identify high-affinity peptide ligands in combination with computational design and rational mutagenesis (Dutta et al., 2015; Rezaei Araghi and Keating, 2016). The Keating group has developed peptides from a yeast-surface display library that target Bcl-xL, Mcl-1 or Bfl-1 with high affinity and selectivity (Foight et al., 2014; Rezaei Araghi et al., 2016). Importantly, the most specific peptide targeting Mcl-1 exhibited at least 40-fold specificity over four Bcl-2 homologs. It is encouraging that the high affinity peptide inhibitors for Bcl-2 represent a starting point for further development of peptide-based therapeutics.

Extensive studies have inspired the advances in other novel methods of investigating peptide inhibitors. For example, Gorostizaa and Ernest Giralt group have recently conceived a modular design strategy based on a generalized template (GT) to obtain nano-switchable peptides that can be applied to α-helix mediated PPIs (Nevola et al., 2019). They used the GT peptide approach combining the structural information from the PPI hot spots and finally developed series of light-regulated peptide inhibitors targeting Bcl-xL and Bak. Peptide-based inhibitors following this novel scheme not only retain favorable binding affinity but also possess the capacity to photo-switch in response to light stimulation. It can be applied to fine-tune the precise spatiotemporal regulation of PPIs and representative a novel prospective avenue in drug design and delivery.

The approaches above have been successfully applied to the design of Bcl-2 family PPI inhibitors. In general, in addition to mimicking the PPI hot spots, both the conformational flexibility and spatial arrangement of the peptide-based inhibitors scaffolds are of critical importance for consideration in molecule design. Moreover, novel techniques such as introducing specific cross-linking to rigidize structure and enhance stability against proteolysis are also emerging and expanding our arsenal in targeting PPIs. These successful strategies are instructive for further development of PPI drugs with higher efficiency and selectivity.

### Inhibitors of p53-MDM2

The tumor suppressor gene p53 is known as a human transcription factor that induces cell-cycle arrest and apoptosis, in response to extrinsic stress and DNA damage (Wade et al., 2013). MDM2 and MDMX are considered as negative regulators of p53 through directly binding to its N-terminus and mediate its degradation (Burgess et al., 2016). Amplification of MDM2 and MDMX are frequently observed in tumors harboring wild type p53 (Karni-Schmidt et al., 2016). Numerous studies have demonstrated that blocking p53-MDM2/MDMX interactions can release p53 from the inhibitory PPI complex and re-activate the p53-dependent cell death (Pazgier et al., 2009; Carvajal et al., 2018). Consequently, development of PPI inhibitors targeting p53-MDM2 interactions has emerged as a promising approach for the treatment of p53-related malignancy.

The p53 binding sites of MDM2 have been determined by X-ray crystallography (Kussie et al., 1996). p53-MDM2 interaction principally depends on the amphipathic α-helix of the p53 and the MDM2 cleft lined with hydrophobic residues. In particular, three key residues in p53, Phe19, Trp23, and Leu26, insert into the MDM2 cleft mediating the proteins interaction. These critical interfacial structures all constitute the structural bases for p53-MDM2 PPI inhibitor discovery.

Peptidomimetic inhibitors featured with different backbones such as β-peptides (Burgess et al., 2016), peptoids (Estrada-Ortiz et al., 2016), and *N*-acylpolyamine (Teveroni et al., 2016) have been extensively studied for targeting p53-MDM2 interactions and they have also been exhaustively reviewed by most of previous excellent reviews (Henchey et al., 2010; Lao et al., 2014; Teveroni et al., 2016). On the other hand, unnatural peptide scaffolds or frameworks such as γ-AApeptides mimicking the secondary structure of native p53 peptide are gradually emerging as a new trend for p53-MDM2 PPI inhibitors. Thus, we mainly focus on them and review this novel category of compounds in current section, hoping to supply more insights toward drugging the critical p53-related PPIs as cancer therapeutics.

**Figure 4:**
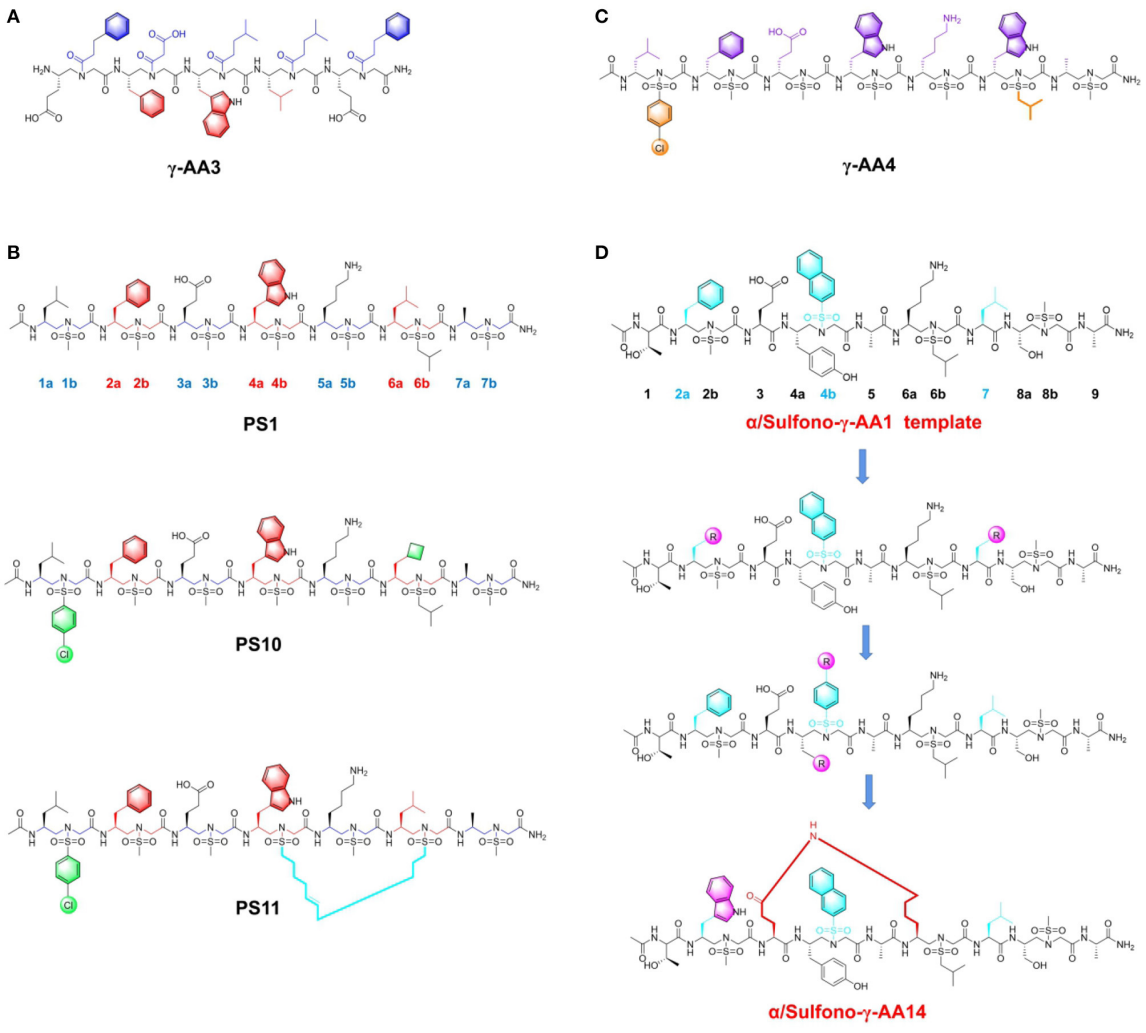
Peptide-based PPI inhibitors targeting p53-MDM2. **(A)** The structure of γ-AApeptide γ-AA3. **(B)** The structure of Sulfono-γ-AApeptide PS1, PS10 and PS11. **(C)** The structre of D-Sulfono-γ-AApeptide γ-AA4. **(D)** The scheme of chemical modifications for α/Sulfono- γ-AApeptides.

Niu et al. (2011) first proposed strategy for synthesis of several γ-AApeptide sequences with diverse side functional groups, which compared to canonical p53 mimicry peptide, display higher potency to inhibit p53-MDM2 interaction. They initially designed unique γ-AApeptide sequences based on the key Phe19, Trp23 and Leu26 residues from p53 implicated in PPI with MDM2 (Niu et al., 2011). In the synthetic route, γ-AApeptide building blocks were generated on a solid phase by adapting monomer building block strategy. ELISA results indicated that **γ-AA3** (Figure 4A), as a new type of peptide mimic with an IC_50_ of 50 μM, is capable of disrupting p53-MDM2 interaction. Further computational modeling unveiled that the side chain moieties from **γ-AA3** overlap well with Phe19, Trp23, and Leu26 of the p53 helical domain, thereby conferring its inhibitory effect. Significantly, the **γ-AA3** showed outstanding resistance to enzymatic degradation as demonstrated by HPLC monitoring. These results suggested that γ-AApeptides are a series of promising lead compounds, expanding the peptidomimetics family in the application of blocking p53-MDM2 interaction.

Subsequently, the research priority is to further enhance inhibitory potency based on current frameworks. The investigators envisioned that previous bioactive peptides could not readily form the stable helical formation to fully mimic the interfacial structure of p53. Considering this regard, sulfonamide groups were introduced to the existing γ-AApeptides to induce the scaffold bending and increase their folding propensity (Teng et al., 2018). Consequently, sulfono-γ-AApeptides, the subclass of γ-AApeptides, were constructed to better simulate the p53 α-helical conformation (Sang et al., 2020b). This series of peptides are stabilized by the intramolecular hydrogen bond interaction, which promotes their formation of α-helix conformation in solution, thereby exhibiting enhanced PPI blocking efficacy. The researchers next designed sulfono-γ-AApeptides featured with chiral side chains at positions 2a, 4a, and 6a, which are identical to the crucial side chains of Phe19, Trp23, and Leu26 in p53. The first sulfono-γ-AApeptide sequence **PS1** (Figure 4B), exhibited IC_50_ and Kd values of 3.95 μM and 98 nM toward MDM2. Then they fixed three key positions, including 2a, 4a, and 6a, and tried to modify the functional groups of other side chains in the sequence. When changing the methyl group to the cyclobutylmethyl group, the best inhibitor **PS10** (Figure 4B), with IC_50_ and Kd values of 0.891 μM and 26 nM was obtained. The computational simulation results indicated that **PS10** shares the similar binding mode of p53 in the hydrophobic cleft of MDM2. The strong binding affinity of **PS10** is due to its intrinsic folding propensity. To improve the cell permeability, they also designed the stapled sulfono-γ-AApeptide **PS11** (Figure 4B), which exhibited remarkable cellular activity.

The above sulfono-γ-AApeptides are left-handed sequences, which attracts the curiosity of researchers to develop the D-sulfono-γ-AApeptide right-handed helical foldamers (Sang et al., 2020a). As the enantiomers of known left-handed L-sulfono-γ-AApeptides, they could be accomplished by the similar strategy for synthesis. As expected, D-sulfono-γ-AApeptide **γ-AA4** (Figure 4C), bearing the replacement of Leu26 with Trp residues, possessed comparable binding affinity to its lead counterpart. Moreover, the peptide **γ-AA4** showed ideal low proteolytic susceptibility, which augments the potential of this new class of D-sulfono-γ-AApeptide in future biological applications.

To expand current scaffolds of peptidomimetic inhibitors and endow more chemical diversity in lead compounds, the investigators selected 1:1 right-handed α/Sulfono-γ-AA peptides as building block monomer for further modification. They chose specific positions 2a, 4b, and 7 of 1:1 α/sulfono-γ-AA peptides to simulate the Phe19, Trp23, and Leu26 in p53 (Shi et al., 2020). Initially, they investigated the effects of side chains containing different substituents on positions 2a and 7 (Figure 4D), which demonstrated that phenyl group and Leu were the optimal moieties at corresponding position. Then they explored the importance of side chain at the position 4b. Classic structural modification strategies were applied to optimize the existing framework, such as changing substituents on the benzene ring or introducing aromatic rings with different sizes. It is demonstrated that these peptidomimetic inhibitors bind more tightly toward MDM2 than the original p53 peptide. Finally, they achieved the most potent helical heterogeneous 1:1 **α/Sulfono-γ-AA1** (Figure 4D), which revealed the binding affinity to MDM2 and MDMX, with Kd of 19.3 nM and 66.8 nM, respectively. It displayed an 18-fold higher binding affinity toward MDM2 than the native p53 peptide. In addition, cross-linking strategy was also utilized into the optimization efforts. Lactam bridge with amide bonds were built to rigidify the main scaffold and improve the cellular inhibitory function. The best stapled peptide **α/Sulfono-γ-AA14** (Figure 4D), with an IC_50_ value of 4.9 μM, was capable of disrupting p53-MDM2 and resisting enzymatic hydrolysis.

All the bioactive peptides, including γ-AApeptide, sulfono-γ-AApeptide, and 1:1 α/Sulfono-γ-AA peptide, show distinctive potency in the inhibition of p53 and MDM2 interaction. Continuous endeavors are devoted to optimizing unnatural peptide backbones, so as to obtain more stable α-helical conformation and higher potent mimicry of regular peptide. The rational design of peptidomimetics targeting p53-MDM2 opens the window for explorations and applications of new class of unnatural peptide sequences toward other therapeutic disease targets.

### Inhibitors of APC-Asef Interaction

Colorectal cancer is one of the major public health threats in modern society, and its pathogenesis involves both genetic and environmental factors. One of the oncogenes for colorectal cancer is Adenomatous Polyposis Coli (APC), whose mutations have been detected in more than 30% of colon cancer patients (Kawasaki et al., 2000). Upon genetic lesions, APC gene will be translated into a truncated form of protein, which binds and constitutively activates its receptor Asef. PPI between truncated APC and Asef relieves Asef from autoinhibition and traps it in an abnormally active state for its guanine nucleotide exchange factor (GEF) activity. It further triggers the downstream oncogenic signals, leading to tumorigenesis and metastasis (Kwong and Dove, 2009; Oh et al., 2018). Thus, the APC-Asef interaction has attracted considerable interests as a highly enticing target for colorectal cancer therapy exploiting PPI inhibitors.

**Figure 5:**
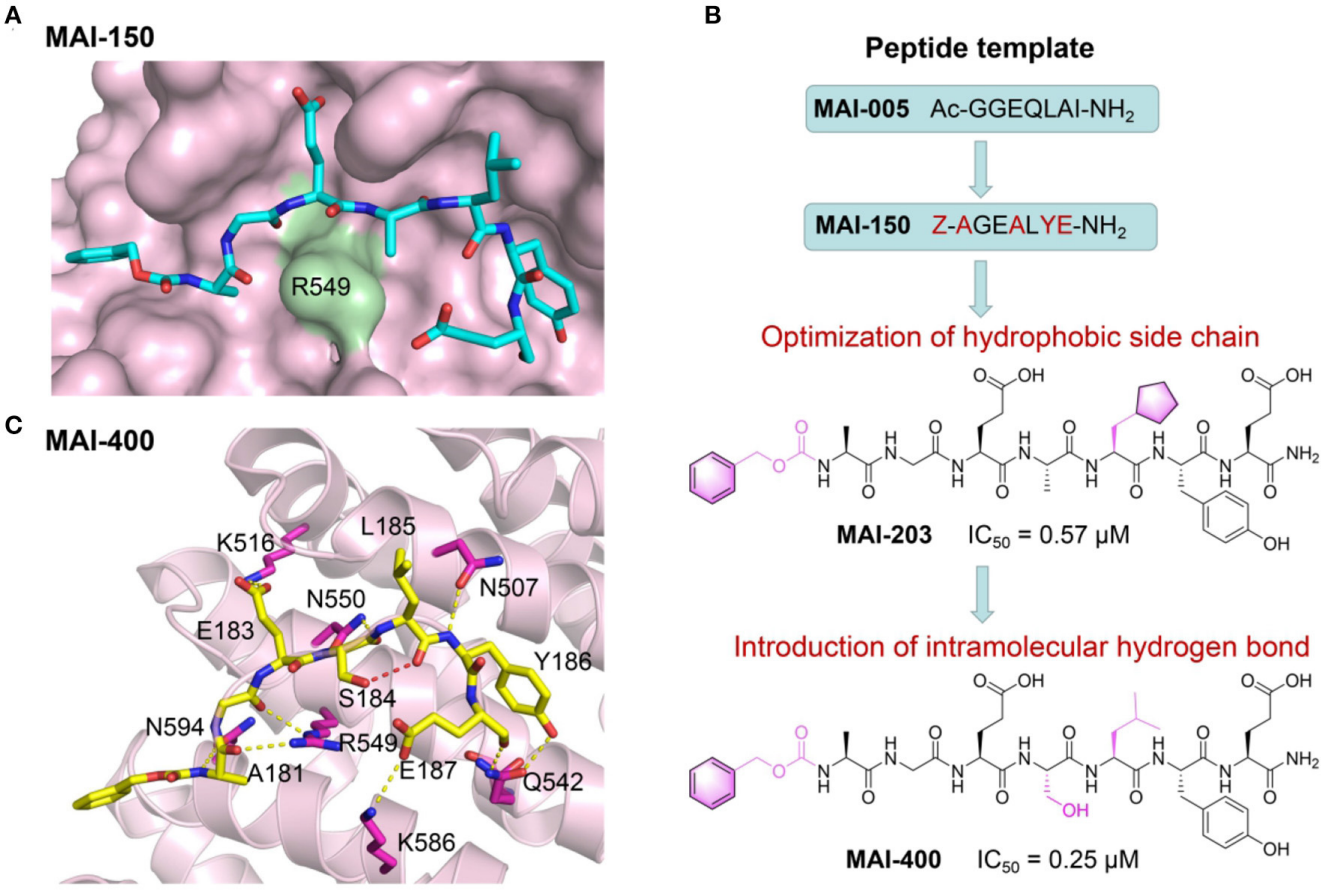
Peptide-based PPI inhibitors targeting APC-Asef PPI. **(A)** Structural overview of MAI150/APC PPI complex (PDB ID: 5IZ6). APC is shown as a solvent-accessible surface (pink), and MAI-150 is depicted by sticks (carbon atoms: cyan). **(B)** Development scheme of MAI analogs as APC-Asef PPI inhibitors. **(C)** Binding interactions between MAI-400 (carbon atoms: yellow) and APC (carbon atoms: pink). The red dashed lines represent the intramolecular hydrogen bonds between MAI-400 and APC.

Zhang et al. have recently reported a series of first-in-class peptidomimetic inhibitors through structure-based rational drug design that potently block the interaction between APC and Asef (Jiang et al., 2017). Based on the previously resolved crystal structure of APC-Asef complex, they identified the segment from Asef as the principal mediator for the PPI. Through mutagenesis and truncated peptide screening, hot-spot residues within for APC-Asef PPI were revealed and a primary peptidomimetics template MAI-005 (^181^GGEQLAI^187^) was designed accordingly. After another round of mutagenesis screening and related crystallography study, a 7-mer peptide library based on the optimized scaffold ^181^AGEAL^185^ was generated. Exhaustive testing with this peptidomimetics library yielded a crucial peptide inhibitor MAI-150 (^181^AGEALYE^187^) with a Ki value of 0.12μM that was 370-fold enhanced compared to the original template MAI-005. Crystallography study revealed that in *apo* state, PPI interface of APC-Asef complex was flat and wide, conferring great difficulties for ligand binding. However, upon inhibitor loading and during its interactions with APC, through “induce-fit” effects, the Arg549 in APC protruded out from the PPI interface and facilitated the formation of a more “druggable” conformation that the peptide inhibitor could bind more easily (Figure 5A). Further cellular and animal model experiments all demonstrated the promising efficacy of these inhibitor peptides. Such study not only reported series of prospective first-in-class peptidomimetics inhibitor targeting the APC-Asef PPI, but also exemplified the possibility of utilizing “induce-fit” mechanistic to tame traditional intractable target. It represented a novel direction for colorectal therapeutics as well as a new avenue for PPI drug discovery.

To improve the inhibitory activity, the researchers carried out another round of modifications based on the aforementioned “druggable” conformation, focusing on the capping groups of the peptidomimetics, especially the side chains of Leu185 and Tyr186 (Yang et al., 2018). Through multiple screening, a cyclopentyalanin-derived peptidomimetic inhibitor MAI-203 was identified as an enhanced compound, which exhibited an eight-fold improvement relative to MAI-150, with a Ki of 0.015 μM and a Kd of 0.036 μM. In order to further optimize the inhibitor peptidomimetics, researchers exploited a rational design strategy of intramolecular hydrogen bonds and introduced such interaction through modifying the lipophilic substituents at the position 185 of MAI-150. As a result, a best-in-class peptidomimetic inhibitor MAI-400 was discovered (Figure 5B). It displayed improved binding affinity toward APC with an IC_50_ of 0.25 μM and a Kd of 0.012 μM. The crystal structure of MAI-400/APC complex revealed that MAI-400 indeed formed an intramolecular hydrogen bond between Ser184 and Leu185. Notably, this hydrogen bond effectively constrained inhibitor's conformation at the binding core along APC surface, contributing to its improved binding and optimized inhibitory performances (Figure 5C).

Collectively, these studies provided a significant example for structured-based design of potent peptidomimetic inhibitors, where through utilizing protein dynamic ensemble and “induce-fit” mechanistic and harnessing rational drug design strategies such as intramolecular hydrogen bond introduction, peptide-based PPI inhibitors could achieved considerable improvement in binding affinity and inhibitory efficacy. Such researches not only shed light on therapeutic agent targeting APC-Asef PPI for colon cancer, but also open a new stream for peptidomimetics PPI inhibitor drug development.

## Discussion and Conclusion

As the pivotal nexus of a broad range of biological and cellular events, PPIs are considered to be one of the utmost important drug target pools in modern medicine. However, historically, this tempting repertoire remains poorly studied and unexplored owing to its unfavorable biochemical and biophysical properties. In the campaign to tame this class of refractory targets, one of the key powerful armory is peptide-based inhibitors, which mimic the PPI binding partners and readily interfere with the PPI systems of pharmaceutical interests. To date, over 60 peptide drugs targeting PPIs have entered into market and clinical applications (Lee et al., 2019), but their development and optimizations still frequently encounter obstacles. One of the vital difficulties in PPI inhibitor discovery is the intractable interfacial structures, which are generally wide and flat and lack well-defined pockets for binding. A potential strategy to tackle this problem is utilizing the induce-fit effect of the target proteins (Nussinov et al., 2014). Upon the inhibitor-protein interactions, the protein structures will be subtly altered, which triggers the surface conformational changes, thereby facilitating the more compact binding of the inhibitors. Importantly, the rapid advances in structural biology and computational biology have enabled the in-depth exploration into the sophisticated conformational ensembles of PPI systems (Qiu et al., 2020; Wang et al., 2021). With the help of techniques such as molecular dynamics (MD) simulations (Yang et al., 2019), the transient intermediate PPI interface conformations can be probed, based on which further structure-based rational inhibitor design can be more easily carried out (Tavakoli and Ganjalikhany, 2019; Yao et al., 2020). Generally, rational design of peptide-based inhibitors toward PPIs depends heavily on reported crystal structures, which can specifically reveal the principles of protein-protein binding modes. It is significant to develop novel molecular scaffolds with an in-depth understanding of the hot spot residues in each interface. In addition, novel peptide-based inhibitors could be more efficiently identified, by combining the computer simulation with chemical structure insights to continuously perform high-throughput screening. For example, SLC (Split-Luciferase Complementation) and HPSEC (high-performance and high-pressure size exclusion chromatography) are recently developed as two novel screening platforms *in vitro*, which have the potential to perform in high-throughput screening of PPI inhibitors. They can overcome the shortcomings of high false positives in regular cell-based methods. SLC was very recently developed by Zhang et al. and through this approach, they have successfully identified two influenza virus PPI inhibitors with broad spectrum antiviral activity (Zhang et al., 2020). As for HPSEC, utilizing the chromatography techniques, it can screen tens of thousands of sequences bearing unnatural amino acids based on size exclusion, achieving higher efficiency and accuracy (Touti et al., 2019). Most of the peptide inhibitors obtained are with high affinity, and most are with nanomolar levels. Besides, chemical modification is indeed an overwhelming strategy to optimize existing molecular scaffolds because the intrinsic properties of PPI inhibitors frequently hamper their successful translation into clinic. Since the PPI partners are mostly large and bulky, their corresponding peptide-based PPI inhibitors are inevitably of relatively large size and high molecular weight, which confer difficulty in their cell membrane permeability and bioavailability. Pharmacological properties and drug-like potential are still required to be optimized in following attempts like hydrocarbon stapling (Chang et al., 2013) and cell-penetrating peptide conjugations (Philippe et al., inpress). Moreover, introduction of D-amino acids, lactam cross-linker or macrocycle (Lautrette et al., 2016; Magiera-Mularz et al., 2017) into the scaffold is beneficial to enhance the metabolic stability (Table 1). Notably, development of small molecules based on the peptide backbone is among the trends in current medicinal studies (Gao et al., 2016; Wang et al., 2019). Without doubt, it is an ever challenging task to design small molecules possessing rigid and hydrophobic features by downsizing the molecular size and mimicking key residues of original peptides (Wang et al., 2019).

**Table 1:** Major obstacles in peptide-based PPI inhibitors design and the representative solutions.

**Obstacles**	**Solutions**
Hydrophobicity	Charged/polar residues incorporation
Stability	Capping additions (acetylation and amidation)
	Cyclization/disulfide bonds
	Hydrocarbon stapled peptides
	D-amino acid replacement
	Unnatural amino acid modifications
	Peptoids
Renal clearance	Macromolecules/polymers conjugations
Permeability	Stapled peptides/cell-penetrating peptides

In summary, with advancement for design techniques, more high-potent and low-toxic peptide drugs could be discovered via rational structure-based design. We could be cautiously optimistic that the next decades will witness new classes of peptide-based inhibitors as promising drug candidates emerging on the market.

## Author Contributions

SL conceived and supervised the project. XW and DN analyzed the results and drafted the manuscript. SL and YL revised the manuscript. All authors discussed the results and reviewed the manuscript.

## Conflict of Interest

The authors declare that the research was conducted in the absence of any commercial or financial relationships that could be construed as a potential conflict of interest.
